# Serratus plane block with sedation for patients submitted to axillary dissection: a prospective case series

**DOI:** 10.1590/0100-6991e-20233398-en

**Published:** 2023-01-24

**Authors:** DANIELE THEOBALD, BRUNO LUÍS DE CASTRO ARAUJO, LUIZ CLAUDIO SANTOS THULER, ROSSANO KEPLER ALVIM FIORELLI

**Affiliations:** 1 - Instituto Nacional de Câncer (INCA), Departamento de Anestesiologia, Hospital do Câncer II - Rio de Janeiro - RJ - Brasil; 2 - Instituto Nacional de Câncer (INCA), Divisão de Pesquisa Clínica - Rio de Janeiro - RJ - Brasil; 3 - Universidade Federal do Estado do Rio de Janeiro (UNIRIO), Programa de Pós-Graduação em Neurologia - Rio de Janeiro - RJ - Brasil; 4 - Universidade Federal do Estado do Rio de Janeiro (UNIRIO), Programa de Pós-Graduação em Medicina - Rio de Janeiro - RJ - Brasil

**Keywords:** Melanoma, Skin Neoplasms, Anesthesia, Conduction, Melanoma, Neoplasias Cutâneas, Anestesia por Condução

## Abstract

Axillary dissection is a standard surgical procedure for stage III skin and soft tissue tumors and is usually performed under general anesthesia. This study aimed to investigate the feasibility of performing axillary dissection with Serratus muscle plane block plus intravenous sedation. Fifteen patients undergoing axillary dissection were prospectively recruited. The patients were evaluated during their pre-operative anesthetic appointment, during their procedure, and at post-operative days 1 and 30. The blockade was performed superficial to the Serratus muscle at the level of fourth rib. Sedation was performed using propofol, fentanyl, dexmedetomidine, and S-ketamine. None of the patients required conversion to general anesthesia. Surgeons showed a highly positive response when asked about the anesthetic technique, and most of them found the technique “indistinguishable” from general anesthesia. The median (interquartile range) pain scores at rest over all time frames was 0 (0-0). Furthermore, no patients developed nausea, hemodynamic instability, or any complications associated with the technique. The Serratus plane block associated with intravenous sedation proved feasible for axillary lymphadenectomy, however, further clinical trials should evaluate potential advantages compared to other techniques.

## RESUMO

A linfadenectomia axilar é um procedimento cirúrgico padrão para tratamento de tumores de pele e partes moles no estádio III e usualmente é realizada sob anestesia geral. A presente serie de casos prospectiva tem por objetivo investigar a viabilidade da realização da linfadenectomia axilar com o uso do bloqueio do plano do músculo serrátil anterior associado a sedação endovenosa. Foram incluídos 15 pacientes no estudo. Os participantes foram recrutados e avaliados durante consulta pré-anestésica ambulatorial, acompanhados durante o dia da cirurgia, no primeiro e no trigésimo dias de pós-operatório. O bloqueio foi realizado anterior ao músculo serrátil anterior ao nível da quarta costela na linha axilar média. A sedação foi realizada com o uso de propofol, fentanil, dexmedetomidina e dextrocetamina. Não houve necessidade de conversão para anestesia geral em nenhum paciente. Os cirurgiões apresentaram resposta altamente positiva quando questionados sobre a técnica anestésica, considerando na maior parte dos casos “indistinguível” da anestesia geral. A mediana (intervalo interquartil) da dor em repouso em todos os momentos avaliados foi 0 (0-0). Além disso, nenhum paciente desenvolveu náuseas, vômitos, instabilidade hemodinâmica ou qualquer complicação relacionada à técnica empregada. O bloqueio do plano do músculo Serrátil anterior associado a sedação venosa se mostrou viável para execução de linfadenectomia axilar, entretanto ensaios clínicos adicionais são necessários para avaliar potenciais vantagens em comparação com outras técnicas.

## INTRODUCTION

Axillary lymphadenectomy is a standard procedure for clinically positive stage III cutaneous melanoma of the upper limbs and trunk. Patients undergoing it are at high risk of postoperative and chronic pain, shoulder functional limitation, and lymphedema, leading to impaired health-related quality of life^1^. Previous studies have evaluated the feasibility of tumescent local anesthesia with sedation for patients undergoing axillary dissection^2^.

Since the description of the ultrasound-guided serratus anterior plane block (SPB) in the last decade by Blanco et al., there has been great interest in the potential of this nerve block for changing outcomes such as opioid consumption and postoperative pain^3^. The aim of this study was to investigate the feasibility of axillary dissection with SPB associated with intravenous sedation.

## METHODS

The protocol was approved by the Ethics in Research Committee of the National Cancer Institute (CEP-INCA), Rio de Janeiro, Brazil (President CHD Silva) on June 2, 2018 (registration number CAAE 89037818.0.0000.5274) registered on the ClinicalTrials.gov on June 25, 2018 (NCT03740815). Specific informed consent for this research was obtained from all participants.

This study consisted of a single-center, prospective case series, described according to the Preferred Reporting Of CasE Series in Surgery (PROCESS) guidelines^4^. We evaluated patients with skin and soft tissue neoplasms who required axillary lymphadenectomy at the National Cancer Institute, Rio de Janeiro. We excluded patients with classification of the American Society of Anesthesiologists (ASA) greater than III, younger than 18 years old, weighing less than 40kg, or with ulceration, infection, or coagulopathy. Cases were non-consecutive due to logistical and human resource limitations stemming from the COVID-19 epidemic, which suspended recruitment for part of 2020. Recruitment began in September 2018 and ceased in February 2021. Patients were evaluated at the outpatient preanesthetic evaluation visit, during the surgical procedure, on the first postoperative day (PO1), and after 30 days (PO30). The research team continued follow-up until PO30.

After standard monitoring, obtaining a peripheral venous line, and initial sedation, SPB was performed between the latissimus dorsi and serratus anterior muscles (superficial approach) at the level of the fourth rib, in the supine position or in lateral decubitus in the operating room. All SPB were performed by the same two anesthesiologists, experienced in regional anesthesia. This reduced the risk of inconsistency between cases. The initial volume was 40ml of a solution of 0.5% ropivacaine, 1% lidocaine, and epinephrine (1:200,000). Five minutes after the block, the research team performed an ultrasound evaluation of the axilla to explore the local anesthetic spread and classified it as poor, fair, good or excellent.

Surgeon and patient satisfaction were also assessed. Immediately after surgery, the primary surgeons rated their satisfaction with the anesthetic technique using a Likert scale such as “extremely satisfied”, “satisfied”, “neither satisfied nor dissatisfied”, “dissatisfied”, and “extremely dissatisfied”. They also compared the technique to general anesthesia. On PO1, we also measured patient satisfaction with the anesthetic technique using the same Likert scale, and the quality of recovery with the 40-item Quality of Recovery scale (QoR-40). Between the postoperative days 28 and 35, we reassessed the participants, with the application of the European Organization for Research and Treatment of Cancer Quality of Life Questionnaire Core 30 (EORTC QLQ-C30), recording the presence of surgical wound infection, skin necrosis, seroma, lymphedema, lymphatic fistula, and date of drain removal.

## RESULTS

We recruited 15 patients, whose baseline characteristics are available in Table 1. Nine patients (60%) were classified as having good or excellent dispersion of the local anesthetic in the ultrasound evaluation. An additional 20ml solution of 1% lidocaine was administered at the discretion of the anesthesia team in five patients with fair or poor axillary local anesthetic distribution. All patients underwent the procedure under moderate to deep sedation with propofol, fentanyl, dexmedetomidine, and S-ketamine. Median operative time was 85 minutes, and no patient required conversion to general anesthesia or any airway device other than an oxygen mask. The median number of lymph nodes extracted was 16 (Interquartile range 12.5-17).

**Table 1:** Baseline clinical and demographic characteristics.

	All patients (n=15)
Age, years	58.8 (10.8)
Male sex	9 (50%)
Skin color	
White	13 (87%)
Non-white	2 (13%)
Weight, kg	83.5 [73.2-96.5]
Height, cm	167.5 (10.7)
Formal education	
<8 years	9 (60%)
≥8 years	6 (40%)
Surgical procedures	
Axillary lymphadenectomy	13 (87%)
Axillary lymphadenectomy and tumor resection	2 (13%)
Arterial hypertension	10 (67%)
Coronary artery disease	2 (13%)
Cerebrovascular disease	1 (6.7%)
Chronic obstructive pulmonary disease	1 (6.7%)
Diabetes	2 (13%)
Smoking	
None	7 (47%)
Previous	4 (27%)
Current	7 (27%)
	All patients (n=15)
ASA Physical State Classification	
I	1 (6.7%)
II	9 (60%)
III	5 (33%)
ECOG performance rating	
0	7 (47%)
1	8 (53%)
Revised cardiac index, class	
I	12 (80%)
II	2 (13%)
III	1 (7%)
Laboratory parameters	
Hemoglobin, g/dL	14.16 (1.05)
Creatinine, mg/dL	0.9 (0.2)
Glucose, mg/dL	98.0 [94.5-115.5]
Albumin, mg/dL^a^	4.54 (0.28)
Histological type ^b^	
Melanoma	12 (80%)
non-melanom^a^	3 (20%)
Melanoma staging^b^	
IIIa	1 (8.3%)
IIIb	3 (25%)
III	6 (50%)
IIId	2 (17%)
Non-melanoma staging	
III	2 (67%)
IV	1 (33%)

Data are presented as n (%), mean (standard deviation) and median [interquartile range]. ASA: American Society of Anesthesiologists ; ECOG: Eastern Cooperative Oncology Group . ^a^Data from 13 cases; ^b^Staging was stratified by histological type and grouped according to the 8^th^ Edition of the American Joint Committee on Cancer Manual.

**Table 2:** Postoperative data.

	All patients (n=15)
Intraoperative data	
Features of the serratus anterior muscle Plane block	
Ropivacaine dose, mg	200 [200-200]
Dose of Lidocaine, mg	400 [400-400]
Volume	40 [40-60]
Axillary dispersion, USG	
Excellent	6 (40%)
Good	3 (20%)
	All patients (n=15)
Fair	3 (20%)
Poor	3 (20%)
Systemic anesthetics in the OR	
Propofol, mg	922.5 [706.8-1031.8]
Fentanyl, mg	0.070 [0.060-0.080]
Ketamine, mg/kg	0.59 [0.36-0.72]
Dexmedetomidine, µg/kg	1.46 [0.89-1.59]
Total time, min	
Block execution	10 [6.5-12.5]
From block to incision	20 [16-27.5]
Surgery time	85 [77.5-91.5]
In OR	140 [137.5-150]
At PACU	124 [120-134]
Use of analgesics at the visit on the first postoperative day	
Non-controlled analgesics	
Dipyrone	13 (87%)
Paracetamol	1 (6.7%)
None	1 (6.7%)
Oral morphine equivalents	
0	13 (87%)
40	1 (6.7%)
50	1 (6.7%)
Late postoperative data	
Number of lymph nodes	16 [12.5-17]
Lenght of stay	1 [1-1]
Seromaa	3 (23%)
Lymphedemaa	2 (15%)
Lymphatic fistulaa	1 (7.7%)
Wound infectiona	3 (23%)

^a^Data from 13 cases available; Data are presented as n (%) and median [interquartile range]; OR: Operating Room; PACU: Post-Anesthetic Care Unit; USG: Ultrasound.

**Table 3:** QoR-40 and EORTC QLQ-C30 scores - Difference between pre- and postoperative values.

	Preoperative	Postoperative	Mean difference (CI)	p-value
QoR-40^a^	181.7 (12.1)	190.0 (6.6)	8.27 (1.75-14.78)	0.017
EORTC QLQ-C30^b^				
Overall health status / QL	76.9 (23.1)	78.2 (13.4)	1.3 (-14.4-16.9)	0.861
Functional scales				
Physical capacity	82.0 (15.5)	81.0 (17.0)	-1.0 (-11.1-9.1)	0.829
Functional capacity	83.3 (21.5)	75.6 (21.1)	-7.7 (-20.4-5.1)	0.213
Emotional capacity	69.2 (22.7)	78.2 (23.5)	9.0 (-6.5-24.4)	0.230
Cognitive ability	82.1 (17.3)	88.3 (15.2)	1.3 (-5.2-7.7)	0.672
Social ability	88.5 (14.2)	89.7 (14.5)	1.3 (-9.9-12.5)	0.809
Symptom scales				
Fatigue	14.5 (20.0)	17.9 (16.1)	3.4 (-7.0-13.8)	0.487
Nausea and vomiting	3.8 (7.3)	3.8 (10.0)	0.0 (-7.1-7.1)	1
	Preoperative	Postoperative	Mean difference (CI)	p-value
Pain	11.5 (14.2)	23.1 (23.1)	11.5 (-5.6-28.7)	0.168
Dyspnoea	5.1 (12.5)	5.1 (18.5)	0 (-8.2-8.2)	1
Insomnia	20.5 (21.7)	25.6 (36.3)	5.1 (-16.4-26.7)	0.613
Loss of appetite	10.3 (21.0)	10.3 (21.0)	0 (-8.2-8.2)	1
Constipation	7.7 (14.6)	20.5 (34.8)	12.8 (-2.6-28.3)	0.096
Diarrhea	7.7 (14.6)	0 (0)	-7.7 (-15.5-1.1)	0.082
Financial difficulties	28.2 (38.1)	25.6 (38.9)	-2.6 (-15.5-10.3)	0.672

Preoperative and postoperative scores were presented as mean (SD), mean difference (95% CI) and paired t-test p-value; CI: Confidence Interval; EORTC QLQ-C30: European Organization for Research and Treatment of Cancer Quality of Life Questionnaire Core 30 ; QoR-40: 40-item Quality of Recovery Score ; ^a^QoR-40 was obtained from all 15 patients at the preoperative assessment and on the first postoperative day; ^b^EORTC QLQ-C30 was obtained from all 15 patients at the preoperative assessment and from 13 patients at the 30-day postoperative visit. Only 13 patients with 2 completed consultations were used for statistical analysis.

**Figure 1:**
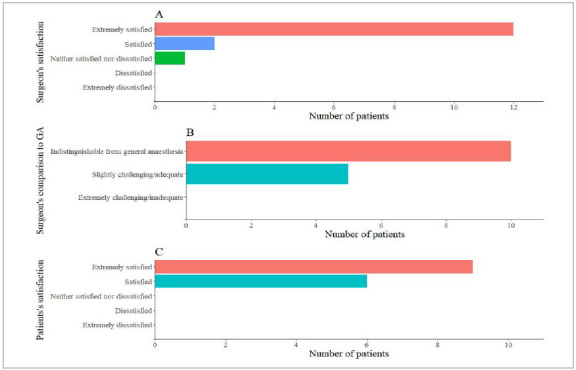
Surgeon’s and patient’s satisfaction scores. GA: General Anesthesia.

The primary surgeon rated the anesthesia on the Likert scale as “satisfied” or “extremely satisfied” on 14 occasions, and “neither satisfied nor dissatisfied”, once. The technique used was classified by surgeons, when compared with general anesthesia, as “indistinguishable from general anesthesia” in 10 cases and “slightly challenging/adequate” in five occasions. On PO1, six patients classified themselves as “satisfied” and nine as “extremely satisfied”. Satisfaction scores are listed in Figure 1.

**Figure 2:**
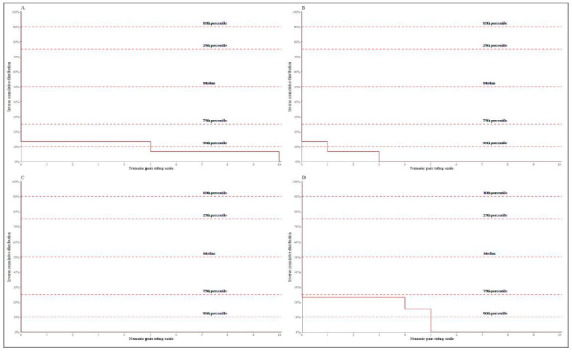
Cumulative distribution of resting 11-item numerical scale pain scores. The x-axis corresponds to the numeric rating scale. The y-axis corresponds to the inverse cumulative distribution of pain scores. The horizontal lines illustrate the 10th, 25th, 50th (median), 75th, and 90th percentiles. Pain scores were recorded (A) upon admission to the Post-Anesthetic Care Unit, (B) 2 hours postoperatively, (C) 24 hours postoperatively, and (D) at the 30-day post-operative visit..

**Figure 3:**
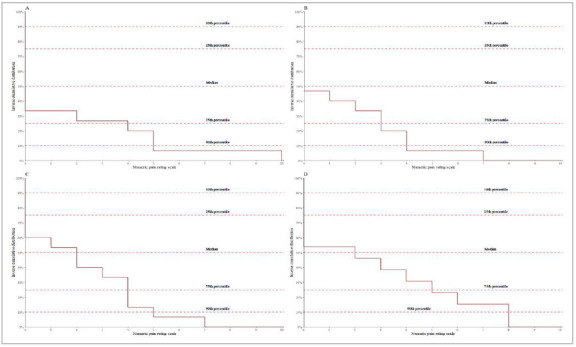
Cumulative distribution of pain scores on the 11-item numeric scale in 90° abduction. The x-axis corresponds to the numeric rating scale. The y-axis corresponds to the inverse cumulative distribution of pain scores. The horizontal lines illustrate the 10th, 25th, 50th (median), 75th, and 90th percentiles. Pain scores were recorded (A) upon admission to the Post-Anesthetic Care Unit, (B) 2 hours postoperatively, (C) 24 hours postoperatively, and (D) at the 30-day post-operative visit..

Pain scores at rest in all evaluated periods had a median of 0 (Interquartile Range 0-0), as described in Figure 2. The distribution of pain scores at 90° abduction is presented as a cumulative distribution in Figure 3. No patient developed nausea, vomiting, dizziness, or hemodynamic instability during the first 24h of follow-up.

The postoperative QOR-40 scores were 8.27 points higher than the preoperative scores (95% CI 1.75-14.78, p=0.017). Two patients did not return to POD30 follow-up. Thus, only 13 patients were available for analysis during this period. None of the EORTC QLQ-C30 domains achieved statistically significant difference between preoperative and postoperative scores.

## DISCUSSION

In a recent systematic review, Araujo et al. compared general anesthesia with other types of anesthesia in surgical procedures for the treatment of malignant melanoma and developed a conceptual framework of potential benefits for choosing regional anesthesia^2^. The elimination of inhalational anesthetics and high-dose opioids, associated with the use of regional anesthesia in appropriate concentrations, may improve early and late clinical outcomes and reduce costs in this population^2^. Additionally, there have been recommendations to avoid general anesthesia during the COVID-19 pandemic. This may affect environmental exposure and ventilator-associated lung injury, possibly impacting the high mortality in the COVID-19 cancer population^5^ ^,^ ^6^.

Few case reports have investigated the use of SPB associated with sedation as an anesthetic technique for surgical procedures in the axilla. Luo et al. described the successful association between SPB at the level of the sixth rib and brachial plexus block via interscalene approach in a patient who underwent resection of a large axillary tumor^7^. Yayik et al. used SPB superficial to the serratus anterior muscle between the fourth and fifth ribs for the resection of a giant axillary lipoma^8^. SPB with 25ml of 0.75% ropivacaine has also been effectively used for lumpectomy and axillary lymphadenectomy^9^. Jajur described a modified SPB between the second and third ribs in the midaxillary line with 10ml of 0.75% ropivacaine associated with adrenaline as effective for a patient undergoing axillary dissection^10^. In another case report, hypnosis and remifentanil techniques were combined with SPB superficial to the serratus anterior muscle to perform a lateral lumpectomy and axillary dissection^11^. Oliveira et al. described the use of SPB and supraclavicular brachial plexus block combined with sedation with dexmedetomidine and ketamine for a patient undergoing debridement of axillary necrotizing fasciitis^12^. Thus, to the best of our knowledge, we describe the first prospective series assessing the feasibility of this technique for patients undergoing axillary dissection.

Based on a cohort study that evaluated data from 2,526 patients who underwent axillary dissection due to stage III cutaneous melanoma, the identification of 10 to 15 lymph nodes in the surgical specimen is consistent with a procedure of adequate quality^13^ The number of lymph nodes obtained in the surgical procedure in the studied sample, the high levels of surgeons’ satisfaction, and the comparability of surgical field conditions with general anesthesia denote the feasibility of performing this surgical procedure with this technique^1^.

The blockade was performed superficially to the serratus anterior muscle in the current series of cases, at the level of the fourth rib. The block thus performed dissects and occupies the axillary lymphadenectomy surgical field, especially facilitating the resection of level I and II lymph nodes. En bloc resection of the surgical specimen and part of the local anesthetic could potentially reduce the duration of the blockade, but also the risk of toxicity. Axillary dispersion measured by anesthesiologists was evaluated as good or excellent (60%), in agreement with a cadaveric study of SPB with 40ml^14^ , and can be improved with other techniques capable of more consistent axillary spread^15^.

## CONCLUSION

The practice of axillary lymphadenectomy using SPB without the combination with general anesthesia has potential economic, organizational, and analgesic advantages to be confirmed in clinical trials.
